# CIDEC Restricts Liver Regeneration by Disturbing Lipid Droplet Triglyceride Turnover

**DOI:** 10.1002/advs.202507048

**Published:** 2025-11-19

**Authors:** Feng Ouyang, Yining Li, Zixuan Zhang, Fangfang Sun, Yihong Shi, Qian Gui, Da Luo, Changyong Hu, Hang Su, Kaiqi Shen, Lu Gao, Hui Yang, Feng‐Jung Chen, Rong Li, Tong‐Jin Zhao, Peng Li, Tian‐Shu Yang

**Affiliations:** ^1^ Shanghai Key Laboratory of Metabolic Remodeling and Health Institute of Metabolism and Integrative Biology Fudan University Shanghai 200438 China; ^2^ Ministry of Education Key Laboratory of Metabolism and Molecular Medicine, Department of Endocrinology and Metabolism Shanghai Key Laboratory of Lung Inflammation and Injury,Department of Pulmonary Medicine Zhongshan Hospital Fudan University Shanghai 200032 China; ^3^ School of Life Sciences Tianjian Laboratory of Advanced Biomedical Sciences Zhengzhou University Zhengzhou 450001 China; ^4^ State Key Laboratory of Membrane Biology and Tsinghua‐Peking Center for Life Sciences Beijing Advanced Innovation Center for Structural Biology School of Life Sciences Tsinghua University Beijing 100084 China

**Keywords:** CIDEC, lipid droplet, liver regeneration, triglyceride

## Abstract

The transient accumulation of triglyceride (TG)‐enriched lipid droplets (LDs) in hepatocytes during early liver regeneration is critical for generation but remains mechanistically unclear, particularly the roles of LD fusion‐associated proteins in lipid mobilization. Here, through integrated lipidomic and transcriptomic analyses, Cell death‐inducing DNA fragmentation factor‐like Effector C (CIDEC), an LD‐associated protein upregulated during this phase is identified, as a negative regulator of regeneration through its unexpected role in sequestering TG within LDs. Mechanistically, CIDEC acts as a metabolic gatekeeper: its depletion after peak LD accumulation promotes TG mobilization and enhances fatty acid oxidation (FAO)‐driven regeneration. This pro‐regenerative effect is abolished by FAO inhibition, underscoring the central role of TG catabolism. Conversely, overexpression of CIDEC or the TG biosynthetic enzyme Diacylglycerol O‐acyltransferase 2 (DGAT2) exacerbates TG retention and impairs liver regeneration. Notably, CIDEC depletion significantly improves regenerative outcomes in mice with chronic steatosis. These findings reveal a previously unrecognized role for LD fusion in regulating the TG storage‐utilization balance, where its suppression promotes metabolic flexibility to meet the energetic demands of liver regeneration. This metabolic checkpoint may be targeted to overcome impaired liver regeneration associated with fatty liver disease.

## Graphical abstract

This study identifies CIDEC, an lipid droplet (LD)‐fusion associated protein upregulated by CREBH during early regeneration, as a negative regulator of liver regeneration through its unexpected role in sequestering triglyceride (TG) within LDs. CIDEC depletion reduces LD size, mobilizes LD‐stored TG, enhances TG catabolism and free fatty acid (FFA) oxidation, and improves liver regeneration.

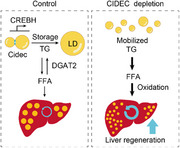

## 1 Introduction

The liver, the body's largest internal organ, possesses a remarkable ability to regenerate after injury. This process requires extensive metabolic adjustments to support cell proliferation and tissue repair. One prominent change is the transient accumulation of large amounts of lipids, particularly triglyceride (TG), in lipid droplets (LDs) during the early phase of regeneration.^[^ ^1^, ^2^ ^]^ Although several studies collectively suggest a requisite role for LD deposition in liver regeneration, a clear understanding remains elusive.^[^ ^3^ ^]^ These conflicting findings may stem from the highly dynamic nature of LD deposition, characterized by transient accumulation that rapidly declines after reaching its peak.^[^ ^4^, ^5^ ^]^ Such precisely timed fluctuations strongly imply the existence of coordinated regulatory mechanisms governing both lipid sequestration into and release from LDs. Compared to lipid sequestration, lipid mobilization may play a more crucial, yet often overlooked, role in liver regeneration. LD‐associated proteins are likely key players in coordinating lipid metabolism to support regeneration. For example, mice deficient in the LD protein adipose differentiation‐related protein (ADRP/Plin2) exhibit reduced hepatic TG accumulation and impaired liver regeneration following PHx,^[^ ^6^ ^]^ highlighting the importance of LD accumulation in this process. However, the precise mechanisms governing lipid mobilization and the roles of LD‐associated proteins in liver regeneration still remain unclear.

Cell death inducing DNA fragmentation factor‐like Effector C (Cidec), also known as fat‐specific protein 27 (FSP27) in mice, is a LD‐associated protein essential for lipid storage in adipocytes.^[^ ^7^, ^8^ ^]^ Research from our group and others has shown that CIDEC promotes LD growth by facilitating lipid transfer at LD contact sites.^[^ ^9^, ^10^, ^11^, ^12^, ^13^ ^]^ In the liver, an alternative Cidec isoform called Cidecβ is expressed, which contains an additional ten amino acids at the N‐terminus of the original Cidecα isoform.^[^ ^14^ ^]^ Transcription of *Cidec* in the liver is regulated by the transcription factor cAMP‐responsive element‐binding protein H (CREBH, also known as CREB3L3), a member of the CREB family that is specifically expressed in the liver, small intestine and stomach.^[^ ^14^ ^]^ Studies in murine models revealed a significant role of CIDEC in hepatic steatosis.^[^ ^14^, ^15^, ^16^ ^]^ However, its role in liver regeneration, particularly in regulating lipid metabolism, has yet to be elucidated.

Here, we explored the role of CIDEC and its involvement in TG mobilization from LDs during liver regeneration. We found that CIDEC depletion in hepatocytes enhances liver regeneration, whereas overexpression of CIDEC results in impaired liver regeneration. Concurrently, CIDEC loss leads to enhanced TG turnover and mitochondrial fatty acid oxidation (FAO). Importantly, inhibition of FAO abrogated the beneficial effect of CIDEC depletion on liver regeneration. Additionally, promoting TG synthesis impaired liver regeneration, indicating that an imbalance between TG storage and utilization contributes to defective regeneration. Notably, CIDEC depletion improved liver regeneration in mice with HFD‐induced chronic steatosis. Our observations imply that CIDEC predisposes to TG storage during liver regeneration, which hinders TG turnover and the liver's ability to regenerate efficiently following injury.

## 2 Results

### 2.1 Lipidomic and Transcriptomic Reprogramming During Liver Regeneration

**Figure 1:**
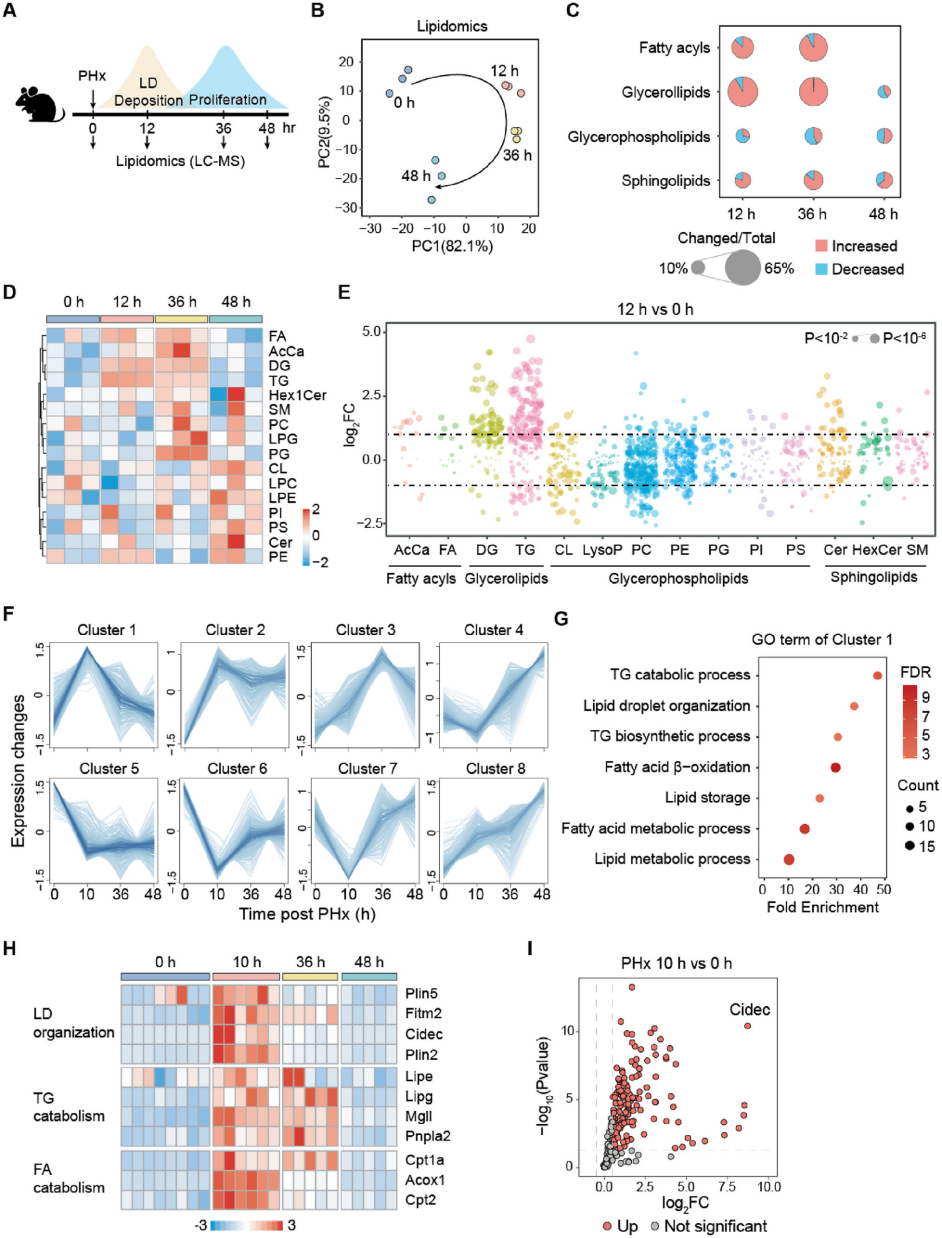
Lipidomic and transcriptomic reprogramming during liver regeneration. A) An overview of experimental design. Livers from mice were collected at 0, 12, 36, and 48 h post partial hepatectomy (PHx) and then extracted for lipidomic analysis. *n* = 3/group. Results were shown from B–E). (B) Principal components analysis (PCA) plots based on the relative abundance of lipid species. (C) Pie charts depicting the proportion of increased and decreased lipid species in each lipid category, showing changes in livers collected at the indicated time points compared to 0 h. The size of each pie represents the proportion of both increased (red) and decreased (blue) lipids relative to the total lipids in each category. (D) Heatmap of different lipid (sub)classes in liver samples. Each column represents values from an individual animal. (E) Bubble plots of log2 fold changes in abundance of lipid species in livers from mice at 12 h post‐PHx compared to 0 h. F) Mfuzz clustering analysis of 2709 metabolism‐related genes in the GEO dataset GSE95135. G) Gene Ontology (GO) functional enrichment analysis of genes in Cluster 1 from (F). H) Heatmap of selected genes in Cluster 1 from the GEO dataset GSE95135. I) Volcano plot of genes in Cluster 1 (shown in panel F) from livers collected at 10 h post‐PHx compared to 0 h, based on the GEO dataset GSE95135.

During liver regeneration, lipid droplet (LD) deposition peaks ≈8–24 h post‐partial hepatectomy (PHx),^[^ ^4^ ^]^ preceding the peak phase of cell division (24–48 h), indicating that the storage and mobilization of lipids from LDs are dynamic and tightly regulated processes. To investigate lipidomic alterations during and after LD deposition, we carried out a global lipidomic survey of mouse livers following PHx. Liver samples were collected from mice at 0, 12, 36, and 48 h after PHx and analyzed using untargeted lipidomics by ultra‐performance liquid chromatography coupled with mass spectrometry (UPLC‐MS) (**Figure** **1**A). Analysis of the relative abundance of the lipid species revealed significantly shifted lipidomic profiles in livers after PHx (Figure 1B).

A total of 1516 lipid species were identified and categorized into four groups (Figure 1C). We further calculated the number of lipid species with increased or decreased abundance within each category. Among these, fatty acyls and glycerolipids exhibited the most significant changes at 12 and 36 h post‐PHx (Figure 1C). We next compared the summed amounts of the individual lipid classes. The heatmap revealed a significant upregulation of glycerolipids, including diacylglycerides (DG) and triglycerides (TG), at 12 and 36 h post‐PHx (Figure 1D). To further visualize the lipidomic alterations at the peak of LD deposition, a bubble plot was used to compare all detected lipids at 12 h versus 0 h post‐PHx, revealing an upregulation of most lipid species in DG and TG (Figure 1E). These findings highlight a dynamic remodeling of the hepatic lipidome during early liver regeneration, with glycerolipids, particularly DG and TG, undergoing the most pronounced accumulation.

To identify genes associated with lipid remodeling during the LD deposition phase, we applied Mfuzz clustering to 2709 liver metabolism‐related genes, grouping them into eight clusters based on their expression patterns in published transcriptomic datasets (GSE95135) (Figure 1F).^[^ ^17^ ^]^ Among these clusters, Cluster 1 comprises 232 genes that are predominantly upregulated during the LD deposition phase, which corresponds to the early stage of liver regeneration (Figure 1G). Gene Ontology (GO) enrichment analysis of Cluster 1 genes highlighted pathways related to TG catabolism, LD organization, TG biosynthesis, fatty acid β‐oxidation, and lipid storage, indicating that the liver not only stores lipids but also actively catabolizes TG and fatty acids during the LD deposition phase (Figure 1G). A heatmap of genes involved in LD organization, TG catabolism, and fatty acid catabolism revealed that these genes were mainly upregulated in the LD deposition phase of liver regeneration (Figure 1H). Among the genes in Cluster 1, *Cidec* was the most significantly upregulated in the LD deposition phase compared with the normal state (Figure 1I). Collectively, our analysis identifies key genes involved in TG turnover and LD organization, with *Cidec* being notably upregulated during the LD deposition phase of liver regeneration.

### 2.2 Association of CIDEC with TG Turnover and Its Transcriptional Regulation by CREBH

**Figure 2:**
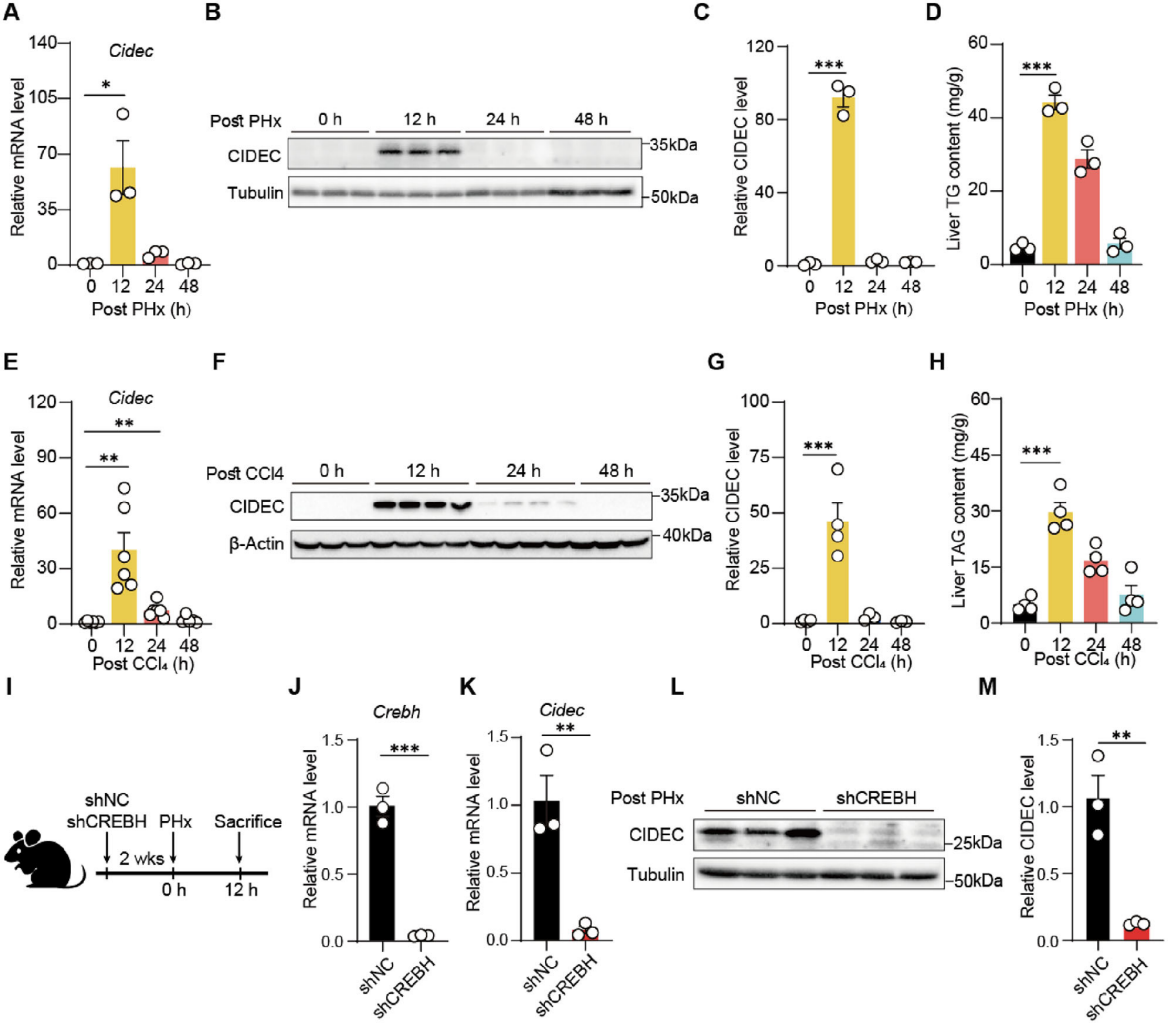
CIDEC expression and transcriptional regulation. A) mRNA expression levels of *Cidec* in mouse liver tissues at the indicated time points post partial hepatectomy (PHx). *n* = 3/group. B,C) Protein levels of CIDEC in liver tissues at the indicated time points after PHx were assessed by immunoblotting (B), and the relative intensities were quantified C). *n* = 3/group. D) Hepatic triacylglycerol (TG) levels at indicated time points after PHx. *n* = 3/group. E) mRNA expression levels of *Cidec* in mouse liver tissues at specified time points after CCl4 administration (1 µL g^−1^ body weight). *n* = 6/group. F,G) Protein levels of CIDEC in liver tissues at the indicated time points after CCl4 administration were assessed by immunoblotting (F), and the ratios to β‐Actin were quantified G). *n* = 3–4/group. H) Hepatic TG levels at indicated time points after CCl4 administration. *n* = 3/group. I) An overview of experimental setup. Mice administered with AAV8 vector encoding short hairpin RNA (shRNA) targeting *Crebh* (shCREBH) or scrambled non‐targeting shRNA (shNC) for 2 weeks were subjected to 70% PHx. Liver samples were collected at 12 h after PHx. J,K) mRNA levels of *Crebh* (J) and *Cidec* (K) in shNC and shCREBH livers. *n* = 3/group. L,M) Protein levels of CIDEC (L) and quantification (M) in shNC and shCREBH liver tissues. *n* = 3/group. Two‐tailed Student's *t*‐test was used to compare two groups, and two‐way ANOVA was used to compare multiple groups. ^*^ *p* < 0.05, ^**^ *p* < 0.01, ^***^ *p* < 0.001. ns, not significant.

To investigate the potential involvement of CIDEC in the regenerating liver, we examined its expression in regenerating liver tissues collected from mice at 0, 12, 24 and 48 h post‐70% PHx. Quantitative PCR (qPCR) and Western blotting showed that CIDEC expression peaked at 12 h post‐PHx, and then rapidly declined to baseline levels, coinciding with the steatotic peak in regenerating livers (**Figure** **2**A–D). To assess whether this expression pattern is unique to surgical resection, we examined CIDEC expression in a carbon tetrachloride (CCl4)‐induced model, in which chemical insult triggers liver injury and regeneration. CIDEC expression peaked at 12 h post‐CCl4 injection (Figure 2E–G), mirroring the changes in liver TG content (Figure 2H). Collectively, these findings suggest that CIDEC expression undergoes a regulated transient increase during liver regeneration, closely associated with lipid turnover.

To investigate the transcriptional regulation of *Cidec* expression in response to liver injury, we performed a motif analysis of genes significantly upregulated genes (log2 fold change > 2, *p* < 0.05) 10 h post‐PHx (data from GSE95135) using HOMER. De novo motif enrichment identified several significantly enriched transcription factors (TFs), including ZFN784, NR2F2, and FOXS1, with CREB being among the top‐enriched TFs (Figure S1A, Supporting Information). Notably, CREBH, a member of the CREB family, has been reported to regulate CIDEC transcription during hepatic steatosis.^[^ ^14^ ^]^ Although CREBH belongs to the CREB family, a specific CREBH motif is not present in standard motif databases. Literature review revealed known CREBH binding motifs and target genes (Table S1, Supporting Information). Subsequent HOMER analysis identified 1000 genes containing these CREBH‐specific binding sites within their promoter regions. These genes were significantly upregulated in livers 10 h post‐PHx compared to untreated livers in the GSE95135 dataset (Figure S1B, Supporting Information). We have further analyzed the expression of reported CREBH target genes. The results showed that *Apoc2*, *Apoa4*, *Apoa5*, *Cidec*, *Fads2, Fgf21*, *G0s2*, and *Pck1* were significantly upregulated, suggesting increased transcriptional activity of CREBH during the early stage of liver regeneration (Figure S1C, Supporting Information).

To demonstrate whether CREBH links liver injury to Cidec upregulation, we depleted CREBH in mouse liver by AAV8 vectors expressing shRNA against *Crebh* (shCREBH) or scrambled non‐targeting shRNA (shNC). 2 weeks after AAV injection, mice were subjected to a 70% PHx (Figure 2I). Injection of shCrebh AAV resulted in a ≈90% reduction of *Crebh* mRNA levels in mouse livers (Figure 2J). Consequently, CREBH depletion led to a significant decrease in CIDEC mRNA and protein levels in the livers of mice 12 h post‐PHx (Figure 2K–M). Additionally, CREBH depletion led to increased liver TG levels 12 h post‐PHx (Figure S1D, Supporting Information), which is probably due to dysregulation of lipoprotein metabolism, consistent with previous study.^[^ ^18^ ^]^ These results indicate that CREBH upregulates transcription of *Cidec* and may disrupt normal lipid processing during the early phase of liver regeneration.

### 2.3 CIDEC Depletion Enhances Liver Repair Following Injury

Given the pronounced upregulation of Cidec alongside LD deposition during early liver regeneration, we hypothesized that CIDEC may play a role in liver recovery. To address this, we generated AAV8 vector encoding short hairpin RNA (shRNA) targeting *Cidec* (shCIDEC) to induce its knockdown in mouse livers. Mice were infused with AAV‐shCIDEC or AAV‐shNC for 2 weeks. shCIDEC mice exhibited a significant reduction in both CIDEC mRNA and protein levels (Figure S2A,B, Supporting Information). Acute CIDEC depletion did not affect body weight, liver size or liver‐to‐body weight ratio (Figure S2C–E, Supporting Information).

**Figure 3:**
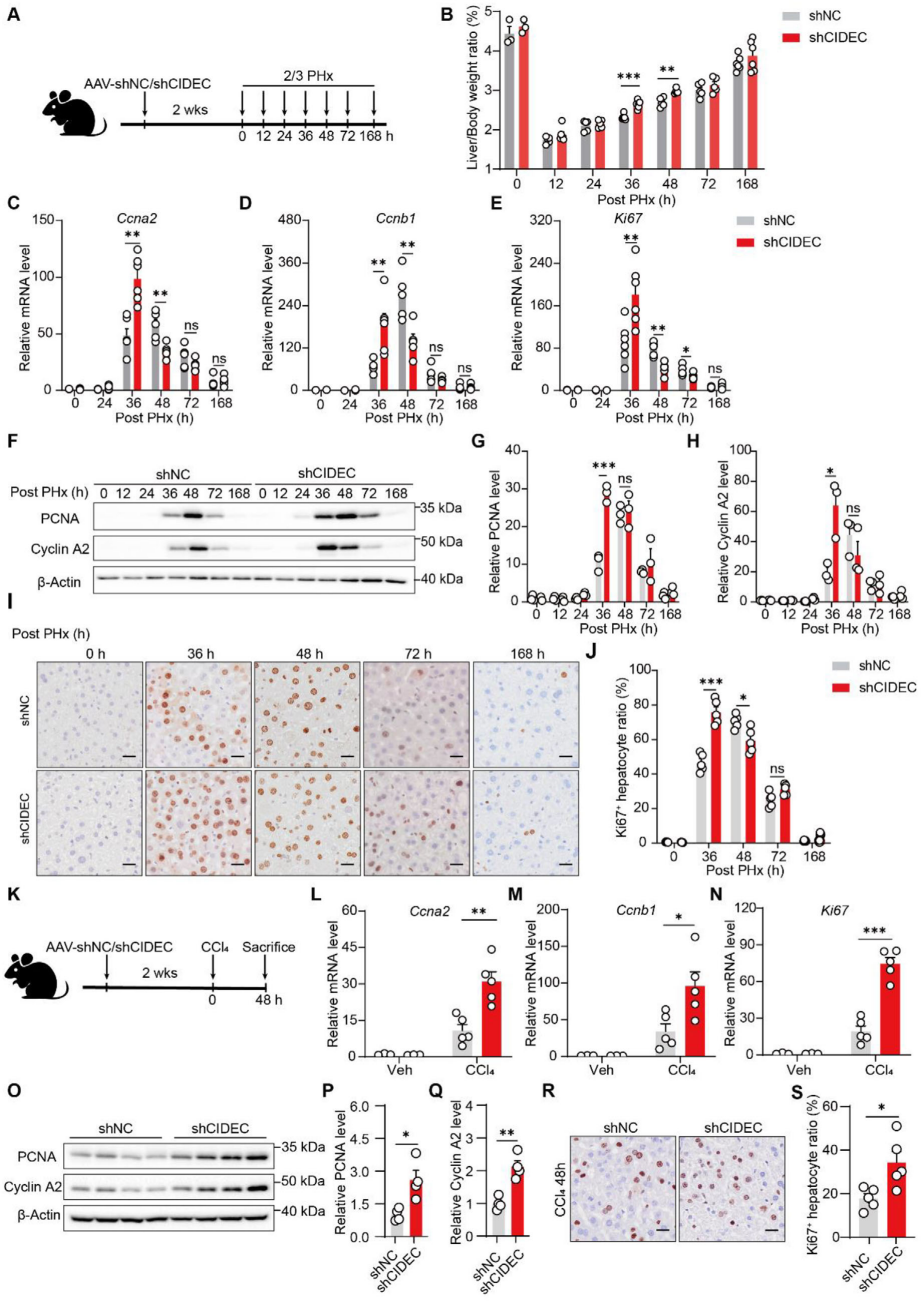
Acute knockdown of CIDEC promotes liver regeneration. A) Schematic overview of experimental setup for partial hepatectomy (PHx)‐induced liver regeneration. Mice administered with AAV8 vector encoding short hairpin RNA (shRNA) targeting *Cidec* (shCIDEC) or scrambled non‐targeting shRNA (shNC) for 2 weeks were subjected to 70% partial hepatectomy (PHx). Liver samples were collected at the indicated time points. B) Liver weight to body weight ratio (LW/BW) at different time points at baseline and post‐PH in mice infused with shNC or shCIDEC. *n* = 3–6/group. C,D,E) mRNA expression levels of *Ccna2* (C), *Ccnb1* (D), and *Ki67* (E) in liver tissues at 36 h post PHx as determined by quantitative PCR. F,G,H) Protein levels of proliferating cell nuclear antigen (PCNA) and Cyclin A2 in liver tissues at 36 h after PHx were assessed by immunoblotting, and the ratios to β‐Actin were quantified (*n* = 3/group). I) Representative immunohistochemistry (IHC) of Ki67 in mice infused with shNC or shCIDEC at different time points. (Scale bars: 100 µm). J) Quantification of Ki67‐positive hepatocytes in shNC and shCIDEC livers at different time points after PHx. K) Schematic overview of the experimental setup in CCl4‐induced liver regeneration. Mice were transfused with shNC or shCIDEC for 2 weeks, followed by CCl4 administration (1 µL g^−1^ body weight), and liver samples were collected 48 h later. *n* = 4–5/group. L,M,N) mRNA levels of *Ccna2* (L), *Ccnb1* (M), and *Ki67* (N) in liver tissues. O) Immunoblotting analysis of Cyclin A2 and PCNA protein levels in liver tissues. P,Q) Quantification of band intensities in (O), with protein levels normalized to β‐Actin. R,S) Representative IHC staining images (R) of Ki67 and its quantification S) in liver sections (*n* = 5/group). Data were expressed as the mean ± SEM with individual values. Scale bar, 100 µm. Two‐tailed Student's *t*‐test was used to compare two groups, and a two‐way ANOVA was used to compare multiple groups. ^*^ *p* < 0.05, ^**^ *p* < 0.01, ^***^ *p* < 0.001. ns, not significant.

To investigate the role of CIDEC in liver regeneration, shNC and shCIDEC mice were subjected to 70% PHx (**Figure** **3**A). As shown in Figure 3B, the remnant liver‐to‐body weight ratios at 36 and 48 h post‐PHx were significantly increased in shCIDEC mice than in shNC mice, suggesting that CIDEC depletion promoted liver recovery after PHx. The transient increase in liver‐to‐body weight ratios in shCIDEC mice at 36 and 48 h post‐PHx, but not at 72 h and thereafter, likely reflects an earlier proliferation peak in shCIDEC livers compared with controls, indicating that CIDEC ablation accelerates liver regeneration. Consistent with this, mRNA levels of cell cycle‐related genes (*Ccna2*, *Ccnb1*, *Ki67*) were significantly elevated in shCidec livers compared to shNC livers at 36 h post‐PHx (Figure 3C–E). In the meantime, protein levels of proliferating cell nuclear antigen (PCNA) and Cyclin A2 were also increased in shCidec livers (Figure 3F–H). Furthermore, the percentage of Ki‐67⁺ cells was higher in shCIDEC livers at 36 h post‐PHx (Figure 3I,J). Hematoxylin and eosin (H&E) images showed that LD accumulation was reduced in shCIDEC livers 24 h post‐PHx (Figure S2F, Supporting Information). These findings suggest that loss of CIDEC promotes hepatocyte proliferation following PHx.

To test whether the inhibitory effect of CIDEC on liver recovery is specific to mechanical injury, shNC and shCIDEC mice were exposed to CCl4 (Figure 3K). As expected, CIDEC depletion significantly upregulated the expression of cell‐cycle‐related genes at 48 h post‐CCl4 exposure (Figure 3L–N). Similarly, protein levels of PCNA and CCNA2 were increased in shCIDEC livers (Figure 3O–Q). Additionally, the proportion of Ki‐67⁺ cells was increased in shCIDEC livers (Figure 3R,S). H&E staining showed that LD accumulation was increased in CIDEC livers 36 h post‐PHx (Figure S2F, Supporting Information). Collectively, these findings suggest that CIDEC depletion enhances liver regeneration following acute mechanical or chemical injuries.

### 2.4 Hepatocyte‐Specific CIDEC Overexpression Impaired Liver Regeneration

**Figure 4:**
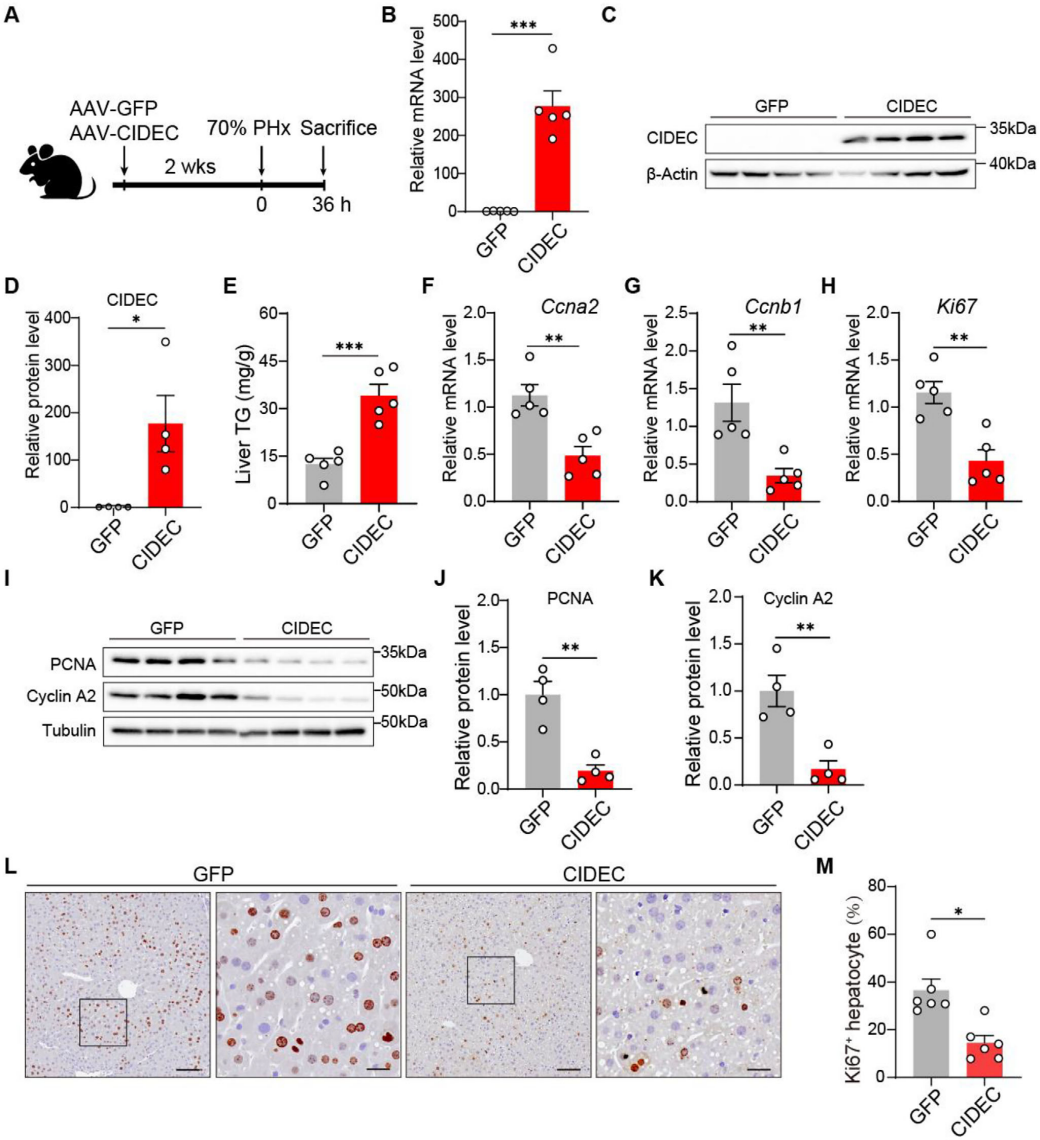
CIDEC overexpression inhibits liver regeneration. A) Schematic overview of experimental design. A 70% partial hepatectomy (PHx) was performed in mice administered with AAV‐GFP or AAV‐CIDEC for 2 weeks. Liver samples were collected 36 h post PHx (*n* = 5). B) mRNA levels of *Cidec* expression in livers from AAV‐CIDEC and GFP groups. C,D) Protein levels of CIDEC in liver tissues at 36 h after PHx was assessed by immunoblotting, and the ratios to β‐Actin were quantified (*n* = 4/group). E) Liver triglycerides (TG) levels at 36 h after 70% PHx. F–H) mRNA expression levels of *Ccna2* (F), *Ccnb1* (G), and *Ki67* (H) in liver tissues 36 h post PHx as determined by quantitative PCR. I–K) Protein levels of proliferation cell nuclear antigen (PCNA) and Cyclin A2 in liver tissues at 36 h after PHx were assessed by immunoblotting, and the ratios to tubulin were quantified (*n* = 4/group). L) Representative immunohistochemistry (IHC) of Ki67 in mice infused with AAV‐GFP or AAV‐CIDEC, 36 h post PHx. Scale bars: 100 µm (left panel) and 25 µm (right panel). M) Quantification of Ki67‐positive hepatocytes per zone in mice infused with AAV‐GFP or AAV‐CIDEC at 36 h post PHx. Data were represented as mean ± SEM. Two‐tailed Student's *t*‐test was used to compare two groups, and two‐way ANOVA was used to compar multiple groups. ^*^ *p* < 0.05, ^**^ *p* < 0.01, ^***^ *p* < 0.001, ns, not significant.

Given that CIDEC depletion promotes liver regeneration, we next investigated whether CIDEC functions as a negative regulator of this process. Notably, CIDEC expression significantly decreased following the peak of LD deposition (Figure 2A–C), suggesting that its downregulation may be necessary in the later phase of liver regeneration and that aberrant upregulation could impair liver regeneration. To test this hypothesis, we exogenously overexpressed CIDEC specifically in mouse hepatocytes using AAV8 vectors driven by the TBG promoter (**Figure** **4**A–D). CIDEC overexpression had no effect on body weight, liver weight, or liver‐to‐body weight ratios before surgery (Figure S3A–C, Supporting Information) but resulted in significantly elevated hepatic TG levels at 36 h post‐PHx (Figure 4E). This finding suggests that CIDEC prolongs lipid deposition and impairs TG turnover during liver regeneration. Concomitantly, liver regeneration was markedly suppressed in CIDEC‐overexpressing mice, as indicated by reduced expression of cell cycle genes, including *Ccna2*, *Ccnb1*, and *Ki67*, at 36 h post‐PHx (Figure 4F–H). In parallel, protein levels of PCNA and Cyclin A2 were diminished, and the number of Ki67‐positive cells was significantly lower compared to GFP‐overexpressing controls (Figure 4I–M). Unexpectedly, an increase in the liver‐to‐body weight ratio was observed in CIDEC‐overexpressing mice, potentially due to enhanced TG accumulation in these livers (Figure S3D, Supporting Information). Histological images further confirmed increased LD accumulation in CIDEC‐overexpressing livers (Figure S3E, Supporting Information). Together, these findings demonstrate that CIDEC overexpression impairs liver regeneration, probably by sequestering TG within LDs and disrupting its timely mobilization.

### 2.5 CIDEC Deficiency Promotes Liver Regeneration Via Enhancing Triglyceride Utilization

**Figure 5:**
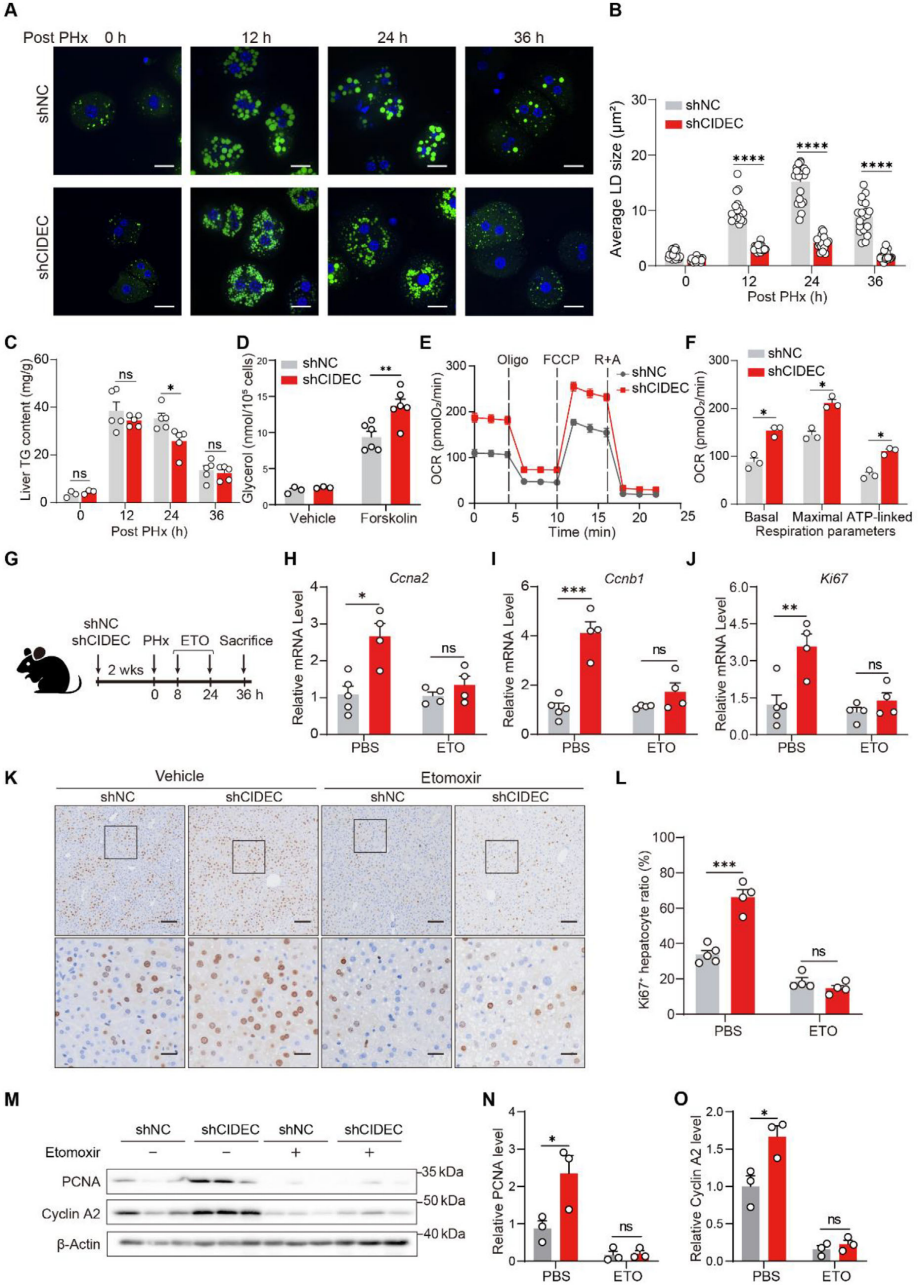
Cidec deficiency promotes liver regeneration via lipid utilization. A,B) Mice administered with AAV8 vector encoding short hairpin RNA (shRNA) targeting *Cidec* (shCIDEC) or scrambled non‐targeting shRNA (shNC) for 2 weeks were subjected to 70% partial hepatectomy (PHx). (A) Primary hepatocytes were isolated at indicated time points and immediately seeded into cell culture dishes. After adhering, they were stained with BODIPY 493/503 and photographed. Scale bars, 20 µm. (B) Statistical analysis of the average lipid droplet (LD) area, corresponding to the images in (A). Each dot represents the average LD size per cell. *n* = 20/group. C) Liver triglyceride (TG) content at the indicated time points were analyzed. (*n* = 5). D) Primary hepatocytes isolated from shNC and shCIDEC mice were treated with 200 µm oleic acid and 100 µm palmitic acid for 16 h, followed by forskolin stimulation to induce lipolysis. Culture medium supernatant was collected for glycerol release. E) Mitochondrial respiration in primary hepatocytes isolated from shNC and shCIDEC mice pre‐treated with 200 µm oleic acid and 100 µm palmitic acid for 16 h. F) The oxygen consumption rate (OCR) at different respiratory stages, including basal, ATP‐linked, and maximal capacity in (E). G) Schematic overview of experimental design. Mice were transfused with shNC or shCIDEC for 2 weeks before undergoing PHx and then treated with etomoxir (5 mg kg^−1^ at 8 h and 10 mg kg^−1^ at 24 h post‐PHx). Liver samples were collected 36 h post‐PHx (*n* = 4–5/group). H–J) mRNA expression levels of *Ccna2* (H), *Ccnb1* (I) and *Ki67* (J) in liver tissues 36 h post PHx. K,L) Representative immunohistochemistry (IHC) of Ki67 (K) and quantification of Ki67‐positive hepatocytes (L) in mice infused with shNC or shCIDEC at 36 h post PHx. Scale bars: 100 µm (top panel) and 25 µm (bottom panel). M–O) Protein levels of proliferating cell nuclear antigen (PCNA) and Cyclin A2 in liver tissues at 36 h after PHx were assessed by immunoblotting (M) and the ratios of PCNA (N) and Cyclin A2 (O) to tubulin were quantified (*n* = 3/group). Data were represented as mean ± SEM. Two‐tailed Student's *t*‐test was used to compare two groups, and a two‐way ANOVA was used to compare multiple groups. ^*^ *p* < 0.05, ^**^ *p* < 0.01, ^***^ *p* < 0.001, ns, not significant.

Given that CIDEC was reported to promote LD fusion and storage in adipocytes, we hence determined the effect of CIDEC depletion on LD storage and mobilization during liver regeneration. We isolated hepatocytes from shNC and shCIDEC mice at various time points after PHx, and assessed LD morphology using BODIPY 493/503. The results showed a marked reduction in the size of LDs in shCIDEC hepatocytes from 12 to 36 h post‐PHx (**Figure** **5**A,B). Conversely, CIDEC overexpression significantly increased LD size compared with GFP controls, especially at 36 h post‐PHx (Figure S4A,B, Supporting Information). Additionally, hepatic TG levels peaked at 12 h and declined more rapidly in shCIDEC livers, as observed at 24 h post‐PHx (Figure 5C). To further elucidate the mechanism, we investigated the impact of CIDEC deficiency on hepatocyte lipolysis. CIDEC‐depleted primary hepatocytes exhibited increased lipolysis (Figure 5D), suggesting that CIDEC depletion promoted TG mobilization from LD stores.

Since TG mobilization and breakdown release fatty acids, we next examined the effect of CIDEC deficiency on fatty acid oxidation (FAO). Oxygen consumption rate (OCR) analysis revealed that CIDEC depletion significantly enhanced basal and maximal respiratory capacity, as well as ATP‐linked respiration, in hepatocytes pre‐treated with exogenous fatty acids (Figure 5E,F). To assess whether CIDEC deficiency promotes liver regeneration via increasing FAO, shNC and shCIDEC mice underwent 70% PHx, with FAO inhibited using etomoxir (ETO), a Carnitine Palmitoyltransferase 1 (CPT1) inhibitor that blocks mitochondrial fatty acid entry (Figure 5G). Inhibition of FAO abrogated the regenerative advantage observed in CIDEC‐depleted mice, indicating that enhanced FAO is essential for the pro‐regenerative effects of CIDEC loss (Figure 5H–O). Collectively, these findings suggest that CIDEC deficiency after peak LD deposition shifts the balance from TG acquisition to catabolism, promoting TG breakdown and enhancing FAO, thereby facilitating liver regeneration.

### 2.6 DGAT2 Overexpression Suppressed Liver Recovery Following Injury

Given our findings that reducing lipid storage by inhibiting LD fusion promotes liver regeneration and conversely, enhancing lipid storage impairs it, we investigated whether directly promoting TG synthesis—the primary lipid stored during LD deposition—could shift the balance from lipid utilization to acquisition, thereby inhibiting liver regeneration. TG synthesis in the liver primarily involves the enzyme Diacylglycerol O‐acyltransferase 2 (DGAT2), which converts DGs into TGs.^[^ ^19^ ^]^ Interestingly, we observed a significant decrease of *Dgat2* expression after surgery or CCl4 administration, which did not recovered until 48 h post‐injury (Figure S5A,B, Supporting Information). As reduced *Dgat2* expression is inconsistent with the increased TG levels observed after liver injury, we also assessed the expression of other key genes involved in TG synthesis (Figure S5C, Supporting Information). Despite reduced *Dgat2* expression post‐liver injury, other key TG synthesis genes were upregulated (Figure S5C–E, Supporting Information). For example, *Agpat3*, *Agpat9*, *Lpin2* showed significant upregulation 10 h after partial hepatectomy (GSE95135) (Figure S5D, Supporting Information),^[^ ^17^ ^]^ and *Agpat2*, *Agpat9*, *Lpin2* were upregulated 8 h after CCl4 administration (GSE167033) (Figure S5E, Supporting Information).^[^ ^20^ ^]^

**Figure 6:**
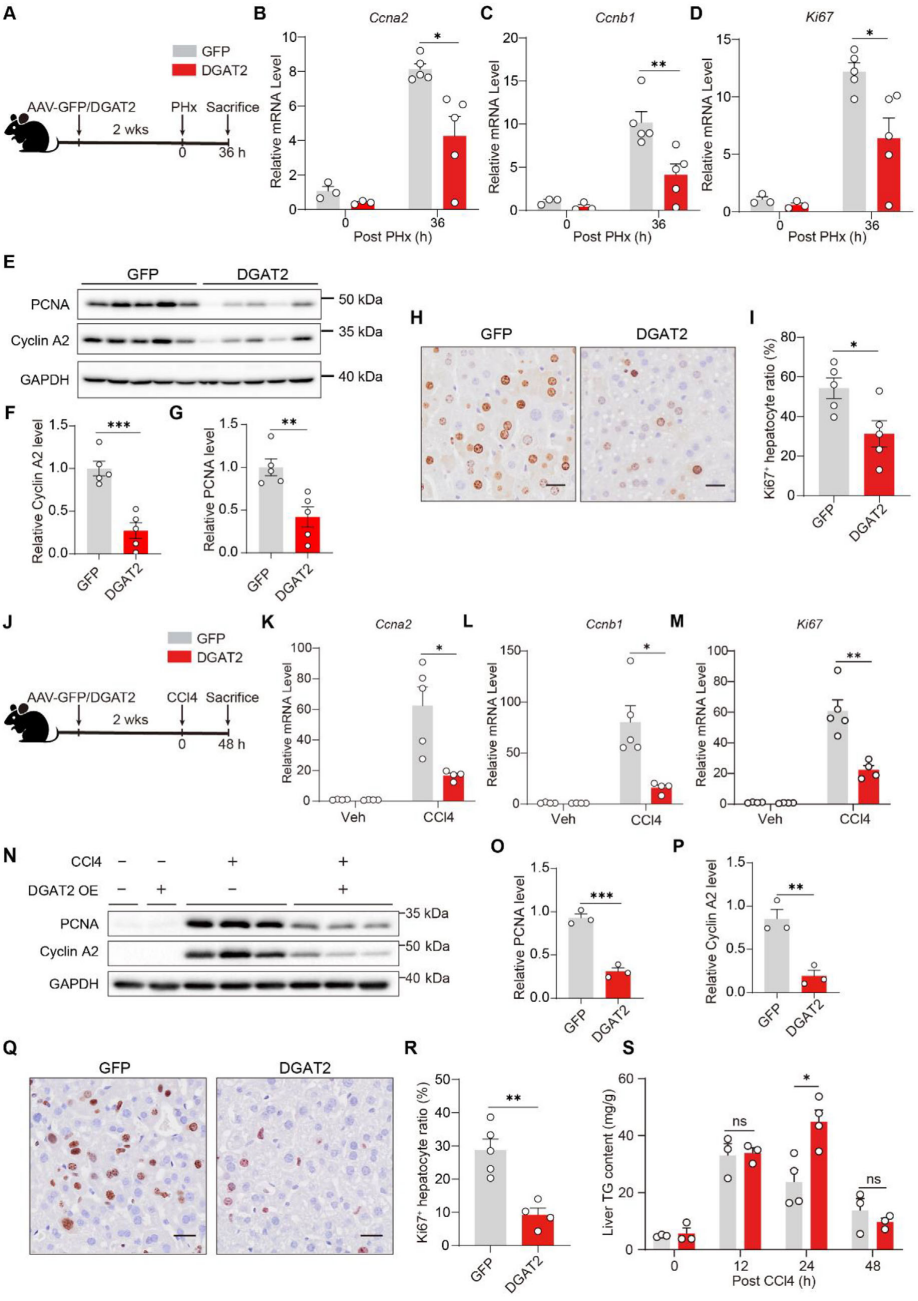
DGAT2 overexpression inhibits liver regeneration. A) Schematic overview of experimental design. Mice administered with AAV‐GFP or AAV‐DGAT2 for 2 weeks were subjected to 70% partial hepatectomy (PHx), and liver samples were collected 36 h later (*n* = 5/group). B–D) mRNA expression levels of *Ccna2* (B), *Ccnb1* (C) and *Ki67* (D) in liver tissues 36 h post‐PHx. E–G) Immunoblotting analysis of Cyclin A2 and PCNA in liver tissues 36 h post PHx treatment and the ratios to β‐Actin were quantified *n* = 5/group). H,I) Representative immunohistochemistry (IHC) of Ki67 (H) and quantification of Ki67‐positive hepatocytes (I) in liver sections from AAV‐GFP or AAV‐DGAT2 group at 36 h post PHx treatment. Scale bars, 25 µm. J) Schematic overview of experimental design. Mice transfused with AAV‐GFP or AAV‐DGAT2 for 2 weeks were administered with CCl4 (1 µL g^−1^ body weight), and liver samples were collected 48 h later (*n* = 4–5/group). K–M) mRNA expression levels of *Ccna2* (K), *Ccnb1* (L) and *Ki67* (M) in liver tissues 48 h post CCl4 treatment. N–P) Immunoblotting analysis of Cyclin A2 and PCNA in liver tissues 48 h after CCl4 treatment and the ratios to GAPDH were quantified (*n* = 3/group). Q,R) Representative IHC of Ki67 (Q) and quantification of Ki67‐positive hepatocytes (R) in liver sections from AAV‐GFP or AAV‐DGAT2 group at 48 h post CCl4 treatment. Scale bars: 25 µm. S) Hepatic triglyceride (TG) levels at indicated time points post‐CCl4 injection in AAV8‐GFP and AAV8‐DGAT2 groups. Data were represented as mean ± SEM. Two‐tailed Student's *t*‐test was used to compare two groups, and two‐way ANOVA was used to compare multiple groups. ^*^ *p* < 0.05, ^**^ *p* < 0.01, ^***^ *p* < 0.001, ^****^ *p*<0.0001 ns, not significant.

As the expression of DGAT2 is decreased following liver injury, we thus suspect that the increased expression of DGAT2 may hinder the expression of cell cycle genes. To test this hypothesis, we exogenously overexpressed DGAT2 specifically in mouse hepatocytes using AAV vectors driven by the TBG promoter (Figure S6A, Supporting Information). Liver regeneration following PHx was suppressed in DGAT2‐overexpressing mice, as evidenced by reduced expression of cell cycle genes, including *Ccna2*, *Ccnb1* and *Ki67*, at 36 h post‐PHx (**Figure** **6**B–D). Concurrently, protein levels of PCNA and Cyclin A2 were diminished, and the number of Ki67‐positive cells was significantly lower compared to GFP‐overexpressing controls (Figure 6E–I). Following CCl4‐induced liver injury, we also observed significantly reduced liver regeneration upon DGAT2 overexpression, as demonstrated by reduced expression of cell cycle genes (Figure 6K–P; Figure S6B, Supporting Information) and decreased percentage of Ki‐67^+^ hepatocytes (Figure 6Q,R). Overexpression of DGAT2 resulted in accumulated TG after the peak of LD deposition, suggesting impaired TG turnover (Figure 6S). Collectively, these results suggest that promoting TG synthesis hinders liver regeneration.

### 2.7 CIDEC Knockdown Improved Liver Regeneration in Animals with Liver Steatosis

**Figure 7:**
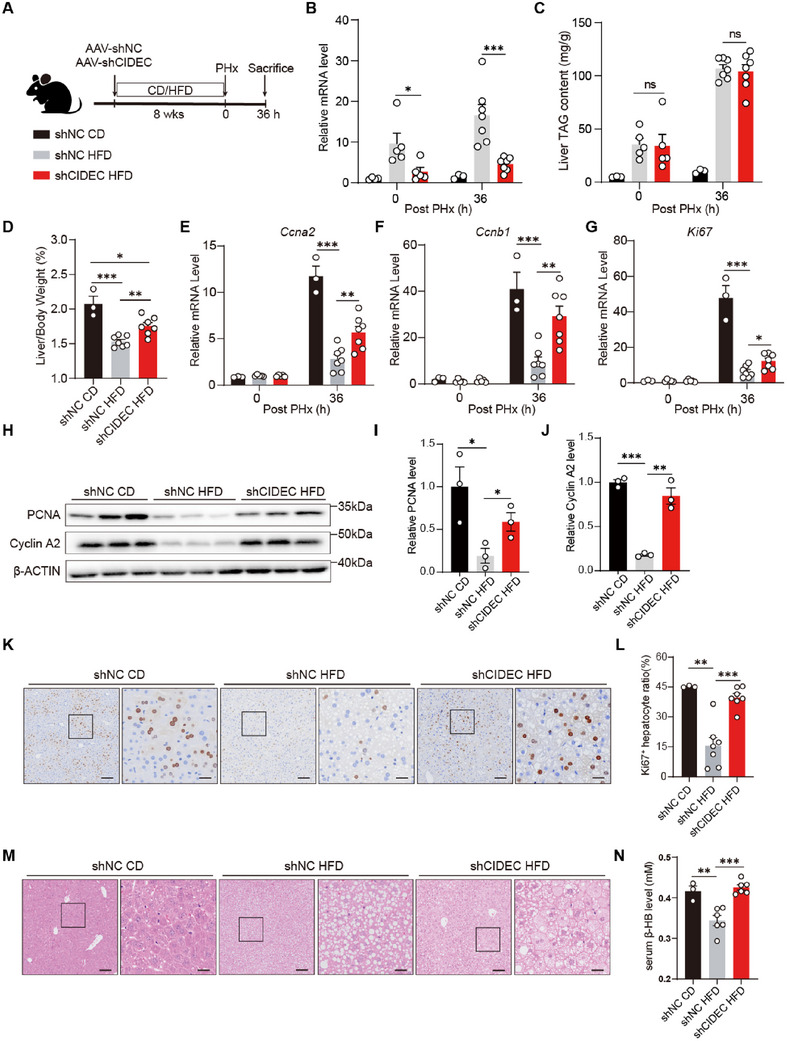
CIDEC depletion improves liver regeneration in animals with chronic liver steatosis. A) Schematic overview of experimental design. Mice received AAV8 vector encoding short hairpin RNA (shRNA) targeting *Cidec* (shCIDEC) or scrambled non‐targeting shRNA (shNC) were subsequently fed either a normal chow diet (CD) or a high‐fat diet (HFD) for 8 weeks. All three groups of mice then underwent 70% partial hepatectomy (PHx) and were sacrificed 36 h post‐surgery (CD shNC, *n* = 3; HFD shNC, *n* = 7; HFD shCIDEC, *n* = 7). B) mRNA levels of *Cidec* in liver tissues from shNC and shCIDEC groups. C) Hepatic triacylglycerol levels in the three groups at 0 and 36 h post PHx. D) Liver‐to‐body weight ratio at 36 h post‐PHx. E–G) mRNA expression levels of *Ccna2* (E), *Ccnb1* (F) and *Ki67* (G) in liver tissues 0 and 36 h post PHx. H–J) Protein levels of proliferation cell nuclear antigen (PCNA) and Cyclin A2 in liver tissues were assessed by immunoblotting (H) and the ratios to β‐actin were quantified (I,J). (*n* = 3/group). K,L) Representative immunohistochemistry (IHC) of Ki67 (K) and quantification of Ki67‐positive hepatocytes (L) in liver sections from shNC or shCIDEC mice at 36 h post PHx. Scale bars: 100 µm (left) and 25 µm (right). M) Representative hematoxylin and eosin (H&E) staining of liver sections from normal chow diet (CD) or HFD‐fed shNC and shCIDEC mice. Scale bars: 100 µm (left) and 25 µm (right). N) Serum β‐hydroxybutyric acid (β‐HB) levels. Data are represented as mean ± SEM. Two‐tailed Student's *t*‐test was used to compare two groups, and two‐way ANOVA was used to compared multiple groups. ^*^ *p* <0.05, ^**^ *p* < 0.01, ns, not significant.

Our previous results suggest the effect of CIDEC deficiency in promoting liver regeneration via lipid utilization. Liver tissues with chronic steatosis are known to have compromised regenerative capacity.^[^ ^21^, ^22^, ^23^, ^24^, ^25^, ^26^, ^27^ ^]^ We therefore determined whether the reduced liver regenerative capacity in chronic liver steatosis associates with CIDEC regulation. Mice infused with AAV carrying shNC or shCIDEC were fed either a normal chow diet (CD) or a high‐fat diet (HFD) for 8 weeks, followed by 70% PHx (**Figure** **7**A). In liver tissues, HFD significantly increased the expression levels of *Cidec* before and after PHx, and shCIDEC AAV treatment significantly suppressed the expression (Figure 7B). CIDEC depletion did not significantly affect liver TG content, body weight or fat mass in HFD‐fed mice (Figure 7C; Figure S7A,B, Supporting Information). In line with previous discoveries, HFD‐fed animals exhibited impaired liver regeneration, as demonstrated by decreased liver‐to‐body weight ratio, reduced expression of cell cycle markers and genes, and less Ki‐67^+^ hepatocytes in comparison with CD animals (Figure 7D–L). And notably, in all the aspects mentioned above, we observed a significant restoration of liver regenerative capacity in CIDEC‐depleted HFD‐fed animals (Figure 7D–L). Importantly, although liver TG levels 36 h after PHx were not significantly different between shCIDEC‐HFD and shNC‐HFD mice (Figure 7C), the size of LDs was notably smaller in the shCIDEC group 36 h after PHx (Figure 7M). We further observed a significant increase in serum 3‐hydroxybutyrate (β‐HB) levels in shCIDEC HFD mice, indicating that liver FAO was upregulated by CIDEC downregulation. Together, these results suggest that CIDEC depletion significantly enhanced liver regeneration in mice with chronic steatosis.

## 3 Discussion

In the early stages of liver regeneration, hepatocytes transiently accumulate large amounts of triacylglycerol in LDs. However, the functional link between transient steatohepatosis and liver regeneration, especially how stored lipids are utilized to support liver regeneration, is not fully understood. In this study, we show that the LD‐associated protein CIDEC inhibits liver regeneration by disrupting LD triglyceride turnover. This highlights the crucial role of LD organization in balancing TG storage and utilization and suggests that CIDEC could serve as a potential therapeutic target for enhancing liver regeneration.

In this study, lipidomic analysis of regenerating livers revealed profound lipidomic reprogramming following liver damage. The levels of TG and DAG remarkably increased 12 h after PHx, suggesting a transient upregulation of TG synthesis and storage during the early regenerating stage. Transcriptomic analysis identified key genes involved in TG turnover, LD organization, and fatty acid β‐oxidation, all of which are notably upregulated during the early phase of liver regeneration. This suggests that the liver actively stores lipids while also catabolizing TG and fatty acids during the peak of the LD deposition phase. Among these genes, *Cidec* is the most upregulated. This upregulation, confirmed by qRT‐PCR and immunoblotting, paralleled transient lipid deposition. Moreover, we demonstrated that *Cidec* upregulation is governed by the liver‐specific transcription factor, CREBH, ensuring its expression is tightly coordinated to the timing of lipid accumulation during liver regeneration.

Although CIDEC has been implicated in promoting LD fusion and lipid storage in adipocytes and hepatocytes, downregulation of CIDEC via shRNA did not affect TG storage at the peak stage. Instead, it unexpectedly accelerated the decline of liver TG content and promoted lipolysis in hepatocytes. These findings suggest that reduced LD fusion and storage enhances TG utilization, highlighting a catabolic role for CIDEC downregulation in regenerating livers. Conversely, overexpression of CIDEC or DGAT2 impaired liver regeneration, suggesting that excessive TG storage disrupts this process, which may result from reduced TG utilization and impaired fatty acid oxidation. Consistent with our findings, animal models with defective β‐oxidation exhibit regenerative defects and persistent LD deposition.^[^ ^4^, ^28^, ^29^ ^]^ Furthermore, we found that CIDEC downregulation was associated with enhanced fatty acid oxidation in shCIDEC hepatocytes. Etomoxir treatment abrogated CIDEC downregulation‐induced liver growth, strongly supporting the role of CIDEC in regulating liver regeneration through lipid utilization and FAO. Collectively, these findings suggest that CIDEC downregulation promotes liver regeneration by shifting lipid storage toward oxidation.

Our study suggests that the dynamic regulation of LDs is crucial for maintaining energy metabolism flexibility, which is vital for supporting liver regeneration. LDs act as both an energy reservoir and a key player in energy mobilization, with a dual function essential for successful regeneration. In the early stages of regeneration, mobilized fatty acids from adipose tissue are rapidly stored in LDs.^[^ ^30^ ^]^ As regeneration progresses, the focus shifts to the efficient mobilization of stored lipids to meet the energy demands in the later phases. Our findings challenge the simplistic view of lipid accumulation as purely beneficial for liver regeneration, highlighting that the key factor is the dynamic turnover of lipids. The findings that CIDEC, which mediates LD fusion, regulates the balance between TG storage and utilization highlight an interesting perspective: LD organization, rather than just TG content, emerges as an independent regulatory dimension regulating lipid turnover and liver regeneration.

The source of lipids during the early stage of liver regeneration remains a critical question. Following PHx, transient hepatic steatosis primarily results from peripheral fat mobilization to the liver. Circulating free fatty acids increase from ≈0.2 to 0.5 mm by 6 h post‐PHx, and whole‐body fat mass drops by ≈20% within 24 h.^[^ ^2^, ^31^, ^32^ ^]^ Lipodystrophic models, such as *fld* and *Seipin* ^−/−^ mice, exhibit reduced transient steatosis and impaired regeneration.^[^ ^33^, ^34^ ^]^ Adipose‐specific deletion of *Lipe* (encoding hormone‐sensitive lipase) halves hepatic TG accumulation and delays regeneration.^[^ ^35^ ^]^ In contrast, liver‐specific deletion of *Fasn* does not alter steatosis or liver regrowth,^[^ ^31^ ^]^ indicating that *de novo* lipogenesis is not a major lipid source in the initial regenerative response. Importantly, our lipidomic analysis (Figure 1D,E) reveals a dramatic increase in substrate availability, including fatty acids and diacylglycerol (DAG), which directly fuels TG synthesis. Studies of DGAT2 enzyme kinetics show that as oleoyl‐CoA and diolein concentrations rise, the enzyme's reaction rate increases correspondingly.^[^ ^36^ ^]^ Thus, the significant increase in substrate availability probably drives the observed rise in TG levels, even with reduced DGAT2 expression.

PHx is a major clinical procedure for patients with primary liver tumors and hepatic metastasis. However, hepatocyte proliferation is significantly impaired in patients with steatosis and in rodent steatosis models following liver resection. ≈30% of patients undergoing liver resection have steatosis, which is associated with a higher risk of complications and mortality.^[^ ^26^, ^27^ ^]^ In rodent models, impaired regenerative capacity has been observed in cases of steatosis, induced by genetic mutations (e.g., leptin‐deficient, leptin‐resistant) or by specific diets, such as high‐fat, high‐fructose, and methionine and choline‐deficient diets.^[^ ^21^, ^22^, ^23^, ^24^, ^25^ ^]^ Consistently, recent studies on therapeutic interventions for metabolic dysfunction–associated steatohepatitis (MASH) have shown that enhancing mitochondrial FAO can restore hepatic metabolic homeostasis and alleviate steatosis.^[^ ^37^ ^]^ As a result, liver tissues with chronic steatosis likely struggle to utilize stored lipids following liver resection or injury. Our findings show that knockdown of CIDEC significantly restored liver regenerative capacity in animals with chronic steotosis, as evidenced by the recovery of proliferation‐related gene expression and the percentage of Ki67‐positive cells, which nearly matched levels seen in CD‐fed mice. Interestingly, CIDEC depletion did not reduce TG content 36 h post‐PHx, but it dramatically reduced LD size and enhanced fatty acid oxidation in the liver. This highlights the critical role of lipid oxidation and utilization during liver regeneration. While further investigation is required to fully elucidate the molecular mechanisms underlying impaired lipid utilization in chronic steatosis, targeting CIDEC offers a promising therapeutic strategy for patients with compromised liver regenerative capacity due to steatosis.

Overall, we have uncovered a mechanism underlying LD organization and TG turnover in liver regeneration, in which the LD‐fusion protein CIDEC suppresses liver regeneration by disrupting TG utilization and FAO, suggesting a potential therapeutic target for improving liver regeneration.

## 4 Experimental Section

### Animal Experiments and Procedures

Male 10 to 12 week‐old C57BL/6J mice were obtained from GemPharmatech (Nanjing, China). Mice were housed under specific pathogen–free conditions at 25 °C with 12 h light/12 h dark cycles, with free access to standard rodent chow and water. All animal protocols were performed with the approval from Fudan University Animal Care Committee (Ethics Approval Number: IDM2024036). Mice were subjected to 70% partial hepatectomy surgery (PHx) as previously described.^[^ ^38^ ^]^ Briefly, the left lateral lobe and the median lobe were surgically removed while the mice were under anesthesia. At indicated time points after surgery, mice were euthanized, and livers were collected for analysis. For the CCl4 model, mice were intraperitoneally injected with CCl4 (289116, Sigma–Aldrich) dissolved in olive oil at a dose of 1.0 µL g^−1^ body weight. CCl4 was diluted in olive oil at a final volume of 50 µL. Control mice received the same dose of corn oil. At 48 h post‐CCl4 administration, mice were euthanized, and livers were collected for histological analysis. For liver regeneration analysis in chronic high‐fat diet (HFD)‐fed mice, 6 to 8 week‐old mice were administrated with adeno‐associated virus 8 (AAV8) vectors and fed with a normal chow diet (CD) (1010088, Jiangsu Synergy Pharmaceutical Bioengineering) or a HFD (60% of energy from fat; Research Diets, D12492) for 8 weeks. AAV injections were performed at the beginning of HFD feeding. To inhibit fatty acid oxidation, mice were intraperitoneally administered with etomoxir (HY‐50202A, MCE) at 5 mg kg^−1^ 8 h post‐PHx and 10 mg kg^−1^ 24 h post‐PHx.

### Adeno‐Associated Virus (AAV)‐Mediated Gene Knockdown and Overexpression

*Cidec* or *Crebh* was silenced using AAV8 carrying murine *Cidec* or *Crebh*‐targeting short hairpin RNA (shRNA) under the liver‐specific TBG promoter, with shRNA sequences listed in Table S2 (Supporting Information). CIDEC or DGAT2 was overexpressed in the liver using AAV8 vectors carrying murine *Cidec*/*Fsp27* or *Dgat2* under the liver‐specific TBG promoter. Gene depletion or overexpression was achieved by administering AAV8 vectors at 2.5 × 10^11^ vector genomes (vg) per mouse. 2 weeks post‐injection, mice underwent 70% partial hepatectomy (PHx) or CCl4 treatment to induce liver regeneration.

### Lipidomic Assay and Analysis

For lipidomic analysis, lipids were extracted from liver samples using the methanol/MTBE‐based extraction method as previously described.^[^ ^39^ ^]^ Liver samples (≈50 mg) was homogenized in 20 × volume of PBS, and then mixed with methanol. After adding 5 mL of MTBE, the mixture was vortexed for 1 min and incubated at room‐temperature for 1 h with shaking, followed by the addition of 1.25 mL water. After centrifugation, the organic phase was collected, dried under nitrogen, resuspended, and analyzed by ultra‐performance liquid chromatography coupled with mass spectrometry (UPLC‐MS) using an Orbitrap Exploris 480 mass spectrometer (Thermo Fisher).

### TG, Glycerol, and Ketone β‐hydroxybutyrate Measurements

Total lipid was isolated from mouse tissue as previously described.^[^ ^40^ ^]^ TG levels in the liver were assessed using a Triglyceride assay kit following the manufacturer's protocol (TR0100, Sigma–Aldrich). Primary mouse hepatocyte were washed three times with PBS and then incubated in phenol red‐free, serum‐free DMEM (31053036, Thermofisher) containing 1% fatty acid‐free BSA (9048‐46‐8, Sigma–Aldrich) in the presence or absence of 10 µm Forskolin (HY‐15371, MCE) for 9 h. The amount of glycerol released into culture medium was measured by free glycerol assay kit (MAK117, Sigma–Aldrich). Serum β‐hydroxybutyrate levels were determined using the β‐hydroxybutyric acid content assay kit (MAK540, Sigma–Aldrich)). All assays were performed according to manufacturer's instructions.

### Isolation of Mouse Primary Hepatocytes

Mouse primary hepatocytes were isolated as described.^[^ ^41^ ^]^ Briefly, 8 week‐old mice were anesthetized, and their livers were perfused with HBSS buffer containing 0.25 mm EGTA for 5 min, followed by a 10 min perfusion with HBSS buffer containing type IV collagenase (0.3 mg mL^−1^) to dissociate the tissue. Cells were filtered and resuspended in William's E Medium (, A1217601, Sigma–Aldrich) containing 10% fetal bovine serum (C04001, VivaCell) and 1% Penicillin‐Streptomycin (C0222, Beyotime). Cells were plated at a density of 2 × 10^5^ cells on collagen‐coated six‐well plates and allowed to recover overnight.

### Oxygen Consumption Analysis of Mouse Primary Hepatocytes

Oxygen consumption rate (OCR) of primary hepatocytes was measured using the OROBOROS Oxygraph‐2K module (OROBOROS Instruments). Cells were pretreated with BSA‐conjugated palmitoylate (100 µm) and oleate (200 µm) overnight before the assay. During OCR measurement, cells were subsequentially treated with oligomycin (0.6 µm), phenylhydrazone (FCCP, 2 µm), and Rotenone (1.25 µm). OCR was normalized to cell number.

### Liver Histology, Immunohistochemical (IHC), and Lipid Droplet Staining

Liver tissues were fixed in ice‐cold 4% paraformaldehyde (PFA) for 24 h, dehydrated, embedded in paraffin, and cut into 5 µm serial cross‐sections. Slides were dried and stained with hematoxylin and eosin (H&E). For IHC staining, tissue sections were dehydrated, and antigen retrieval was performed using sodium citrate. Sections were incubated overnight at 4 °C with primary antibodies, including mouse anti‐Ki67 (1:1000, GB121141, Servicebio), followed by incubation with horseradish peroxidase‐conjugated secondary antibodies (1:200) at 20 °C for 1 h. Sections were then immersed in DAB solution, and the reaction was monitored under a microscope. For lipid droplet staining, primary hepatocytes were isolated from mice at the indicated time points following PHx. After 3 h of cell attachment, the isolated primary hepatocytes were fixed with 4% PFA for 15 min, followed by PBS washing. Cells were then stained with either BODIPY 493/503 or BODIPY 665 dye at a 1:200 dilution for 20 min. After washing, slides were mounted with DAPI‐containing mounting medium. Stained sections were imaged using a Zeiss LSM‐780 confocal microscope, and lipid droplet size per cell was analyzed with ImageJ.

### RNA Isolation and Quantitative Real‐Time PCR (qPCR)

Total RNA of liver tissues were extracted using TRIzol reagent (DP424, TIANGEN Biotech) and reverse transcribed into cDNA according to manufacture's instructions (RK20433, Abclonal). qPCR was carried out using SYBR green Master Mix (11202ES08, YEASEN). Primers were listed in Table S2 (Supporting Information).

### Immunoblot Analysis

Mouse liver tissues were homogenized in RIPA lysis buffer (25 mm Tris‐HCl pH 7.6, 150 mmol L^−1^ NaCl, 1% NP‐40, 1% sodium deoxycholate, and 0.1% SDS) containing protease inhibitors (Roche). Proteins were separated using SDS‐PAGE and probed using antibodies. The following antibodies were used for western blot: anti‐Cyclin A2 (A19036, Abclonal, 1:2000), anti‐PCNA (13 110, CST, 1:4000), and anti‐β‐Actin (1:10 000; 4970, CST). The rabbit polyclonal antibody against mouse CIDEC was generated as previously described.^[^ ^42^ ^]^ Blots were developed and protein levels were quantified using Image Lab Software (Bio‐Rad).

### Analysis of Transcriptomic Data

Publicly available transcriptomic data of liver tissues following PHx were downloaded from the GEO database (GSE95135 and GSE167033). The R version 4.4.1 was used for RNA‐Seq data processing and analysis. A set of 2709 metabolic genes were obtained from the study by Biersoy et al.^[^ ^43^ ^]^ The log2‐transformed reads per kilobase per million (RPKM) value were converted back to the original RPKM data matrix. The average RPKM values were calculated across each time point. Four time points were considered: Control (0 h), Post‐PH (10 h), Post‐PH (36 h), and Post‐PH (48 h). Genes were clustered to based on their expression patterns across these time pints using the fuzzy C‐means clustering algorithm implemented in the R package “Mfuzz” version 2.54.0.^[^ ^44^ ^]^ Gene ontology (GO) enrichment analysis of genes in Cluster 1 was performed using DAVID.^[^ ^45^ ^]^ Motif enrichment analysis was performed using HOMER (v4.12).^[^ ^46^ ^]^ Promoter sequences were defined as 400 bp upstream and 200 bp downstream of the transcription start site (TSS). Target genes containing CREBH‐binding motifs were identified from protein‐encoding genes.

### Statistical Analysis

All data were presented as the mean ± SEM and were statistically analyzed by GraphPad Prism 8.0 software unless otherwise indicated. Student's *t*‐test was used for comparative analysis between two groups, and one‐way ANOVA followed by Tukey's test was used for comparative analysis between multiple groups. *P* values <  0.05 with a 95% confidence interval were considered significant.

## Conflict of Interest

The authors declare no conflict of interest.

## Supporting information



Supporting Information

## Data Availability

The data that support the findings of this study are available on request from the corresponding author. The data are not publicly available due to privacy or ethical restrictions.
