# Supplementary material for: CIDEC Restricts Liver Regeneration by Disturbing Lipid Droplet Triglyceride Turnover
Source: Adv Sci (Weinh). 2025 Nov 19;13(3):e07048. doi: 10.1002/advs.202507048 (PMC12806313; doi:10.1002/advs.202507048)
Supplement: Supplementary file 1 — Supporting Information [file ADVS-13-e07048-s001.docx]

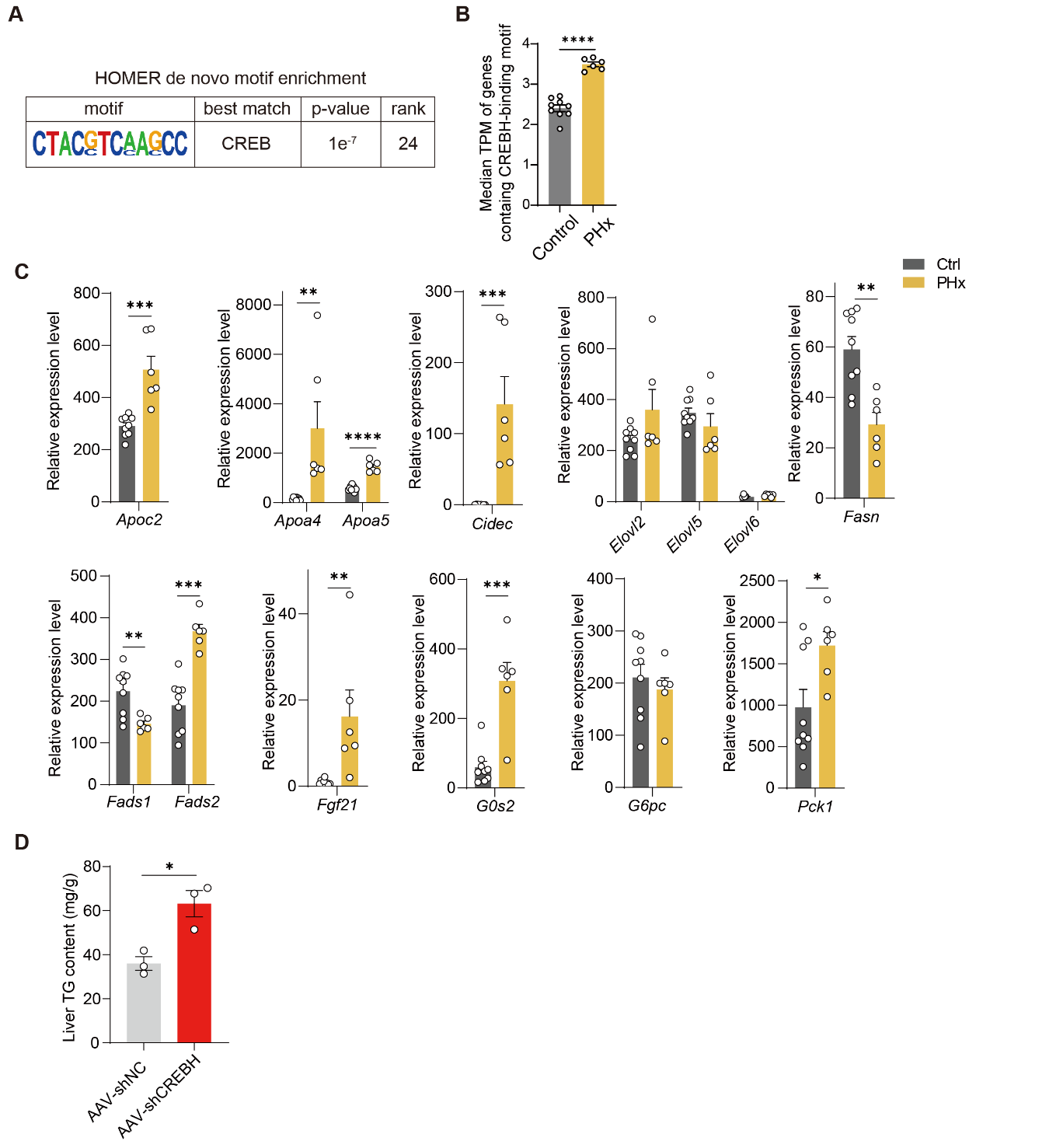


**Figure S****1** **The role of CREBH on lipid metabolism in early stage of liver regeneration.**

(A) HOMER de novo motif analysis of genes significantly upregulated in mouse livers 10 hours post-PHx compared to control livers (data from GSE95135). (B) Median transcripts per million (TPM) of genes containing CREBH-binding motifs in control and 10 hr post-PHx livers (data from GSE95135). (C) TPM levels of reported CREBH target genes. (D) Mice administered with AAV8 vector encoding short hairpin RNA (shRNA) targeting Crebh (shCREBH) or scrambled non-targeting shRNA (shNC) for two weeks were subjected to 70% PHx. Hepatic TG levels were assessed at 12 hours after PHx. Data are presented as mean ± SEM. Two-tailed Student’s *t*-test was used to compare two groups, *p < 0.05, **p < 0.01, ***p < 0.001.

**
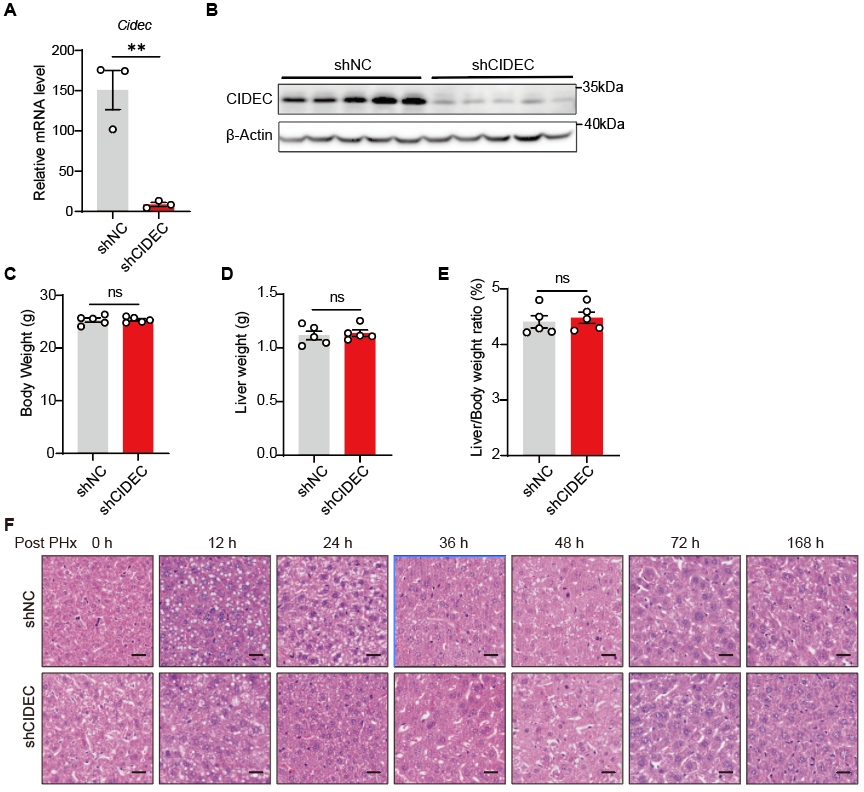
**

**Supplementary Figure S2 Impact of CIDEC knockdown on liver/body weight pre-surgery and histological changes post-surgery.** Eight-week-old mice were injected with AAV8 vector encoding short hairpin RNA (shRNA) targeting Cidec (shCIDEC) or scrambled non-targeting shRNA (shNC) for two weeks. Liver *Cidec* mRNA (A) and protein (B) expression levels in shNC and shCIDEC mice 12 hours after partial hepatectomy (PHx). Body weight (C), liver weight (D) and liver-body weight ratio (E) before surgery were measured. n =5/group. (F) Mice administered with AAV8 vector encoding short hairpin RNA (shRNA) targeting *Cidec* (shCIDEC) or scrambled non-targeting shRNA (shNC) for two weeks were subjected to 70% partial hepatectomy (PHx). Hematoxylin and eosin (H&E) staining of liver tissues collected at indicated time points after PHx were shown. Scale bars, 25 μm. Two-tailed Student’s *t*-test was used to compare two groups. **p < 0.01; ns, not significant.


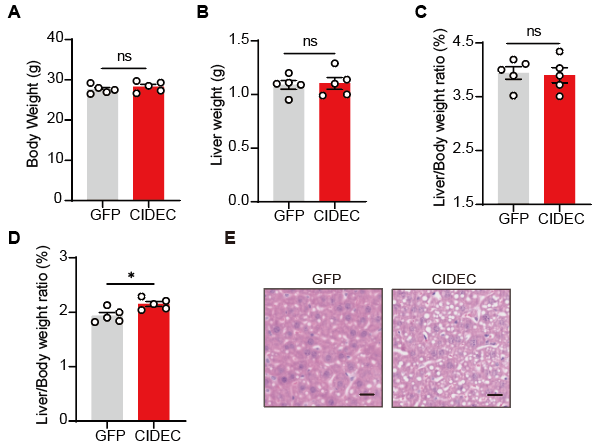


**Figure S3** **Impact of CIDEC overexpression on liver/body weight pre-surgery and histological changes post-surgery.** Eight-week-old mice were injected with GFP and CIDEC adeno-associated virus intravenously. Body weight (A), liver weight (B) and liver-body weight ratio (C) before surgery were measured two weeks later. n =5/group. (D) Liver-body weight ratio 36 h after partial hepatectomy (PHx) in mice injected with GFP and CIDEC AAV. (E) H&E staining of liver tissues collected at 36 h after PHx. Scale bar, 25 μm. Two-tailed Student’s *t*-test was used to compare two groups. *p < 0.05, ns, not significant.


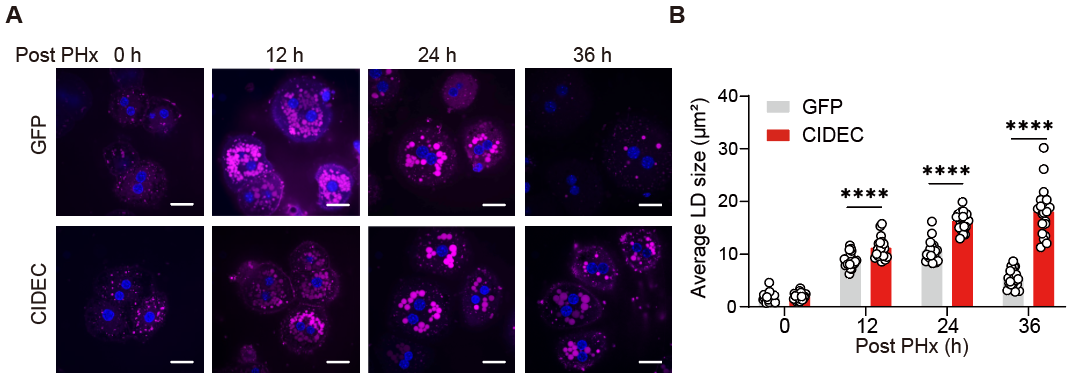


**Figure S4** **CIDEC overexpression increased the size of lipid droplets (LDs) in hepatocytes post partial hepatectomy (PHx).** Mice administered with AAV8 vector encoding GFP and CIDEC for two weeks were subjected to 70% PHx. (A) Primary hepatocytes were isolated at indicated time points and immediately seeded into cell culture dishes. After adhering, they were stained with BODIPY 493/503 and photographed. Scale bars, 20 μm. (B) Statistical analysis of the average lipid droplet (LD) area, corresponding to the images in (A). Each dot represent the average LD size per cell. n = 20/group. Data were represented as mean ± SEM. Two-tailed Student’s *t*-test was used to compare two groups, and two-way ANOVA was used to compared multiple groups. ****p < 0.001.


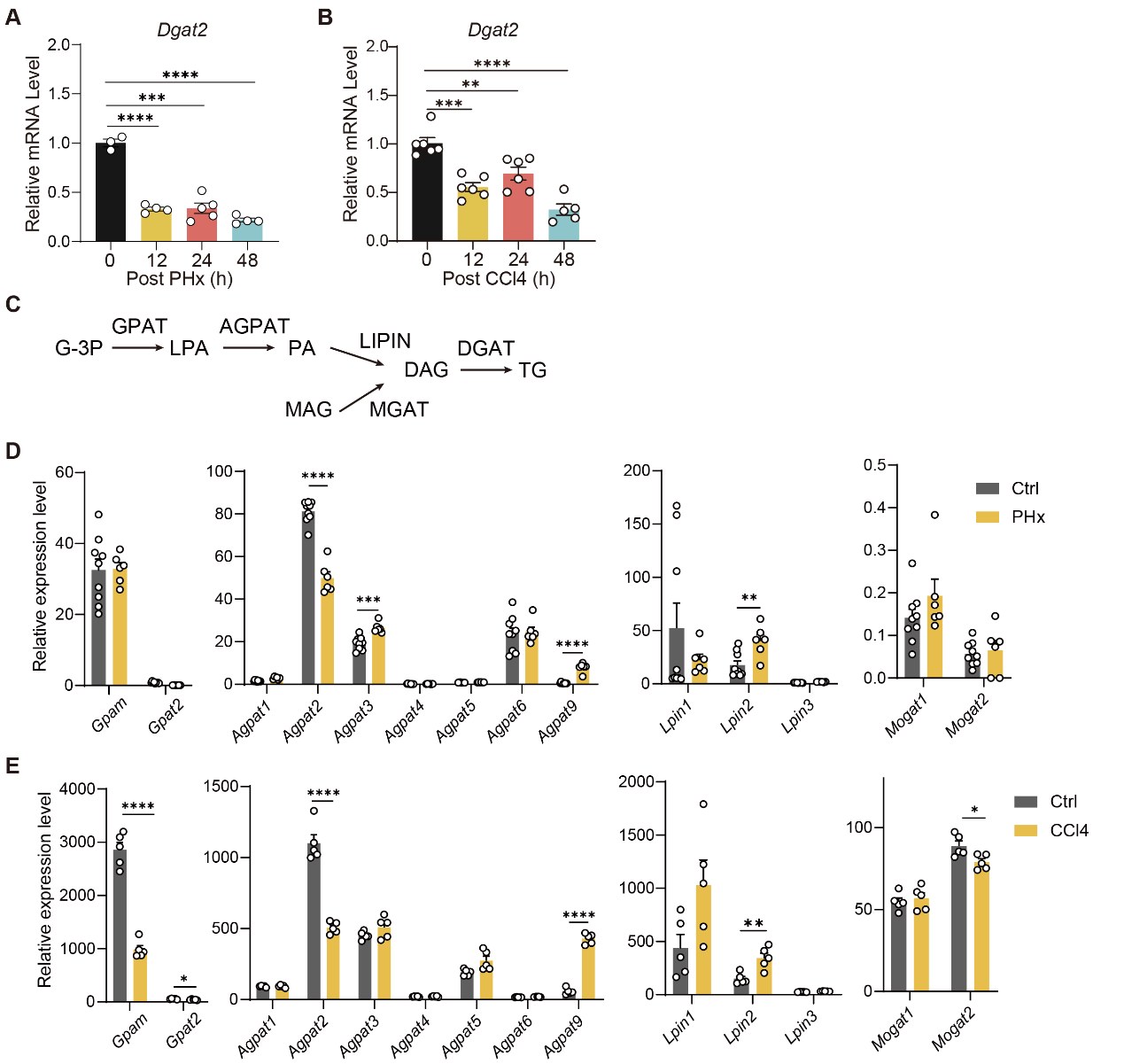


**Supplementary Figure S5** **Expression of DGAT and other genes involved in TG synthesis during the early stages of liver regeneration.** Mice administered with AAV8 vector encoding GFP or DGAT2 for two weeks were subjected to 70% PHx (A) or CCl4 administration (B). The relative mRNA level of *Dgat2* in livers at indicated time points were determined by quantitative PCR (n = 6/group). (C) Triglyceride (TG) synthesis pathway including key metabolites and enzymes. (D) Median transcripts per million (TPM) of TG synthesis genes in liver tissues 10 hours post-partial hepatectomy (PHx) compared to control livers (data from GSE95135). (E) Normalized gene expression levels of TG synthesis genes in liver tissues 8 hours post-CCl4 administration compared to control livers, as determined by gene array (data from GSE167033). Two-tailed Student’s *t*-test was used to compare two groups, *p < 0.05, **p < 0.01, ***p < 0.001, ****p < 0.0001. ns, not significant.

**
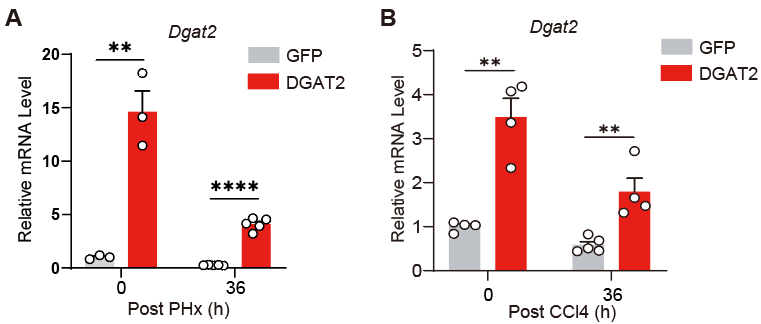
**

**Supplementary Figure S6 DGAT2 over expression in mouse livers pre- and post- liver injury.** Mice transfused with AAV-GFP or AAV-DGAT2 for 2 weeks were subjected to 70% partial hepatectomy (PHx) (A) or CCl4 administration (B), and *Dgat2* mRNA expression levels in liver tissues were measured by quantitative PCR.


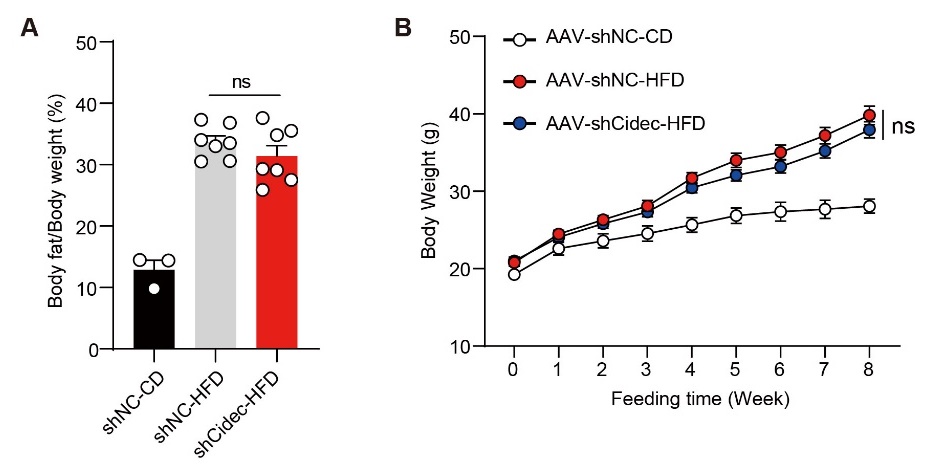


**Supplementary Figure S7** **Effect of CIDEC Knockdown on body fat percentage and weight changes in mice fed high-fat or chow diets. (**A) Body fat percentage in mice fed with a high-fat diet or chow diet for eight weeks. Mice injected with shNC virus or shCIDEC virus had unchanged body fat percentage after feeding a high-fat diet. (B) Body weight changes in mice fed a chow or high-fat diet for eight weeks. Data were expressed as the mean ± SEM with individual values. Two-tailed Student’s *t*-test was used to compare two groups. ns, not significant.

**Supplementary Table S1 Binding sites and target genes of CREBH**

| Binding site | Target gene | Reference |
| --- | --- | --- |
| 5’-ATGACCT-3’  5’-AAGTGGT-3’ | *Pck1*, *G6pc* | ^[1]^ |
| 5’-ACGTGGT-3’ | *Apoa5* | ^[2]^ |
|  | *Acaca, Acacb, Fasn, Apoc2, Apoa4, Elovl2, Elov5, Elovl6* | ^[3]^ |
|  | *Fads1*, *Fads2*, *Elovl2*, *Cidec*, *Apoc2*, *Apoa5*, *Fgf21*, *Apoa4*, *Elov5*, *G0s2* | ^[4]^ |
| 5′-CCACGTTG-3′  5′-CAACGTGG-3′ | *Apoa4* | ^[5]^ |
| 5’-TGACG-3’ | *Fgf21* | ^[6]^ |
| 5’-CAGCGTGA-3’ | *Cidec* | ^[7]^ |

**Supplementary Table S2** Primer sequences of qPCR.

| Primer name | Sequence (5' to 3') |
| --- | --- |
| *36B4*_Forward | ACACTGTGCCCATCTACGAG |
| *36B4*_Reverse | CAGCACTGTGTTGGCATAGAG |
| *Fsp27*_Forward | ATGGACTACGCCATGAAGTCT |
| *Fsp27*_ Reverse | CGGTGCTAACACGACAGGG |
| *Ki67*_Forward | ATCATTGACCGCTCCTTTAGGT |
| *Ki67*_ Reverse | GCTCGCCTTGATGGTTCCT |
| *Ccna2*_Forward | GCCTTCACCATTCATGTGGAT |
| *Ccna2*_Reverse | TTGCTGCGGGTAAAGAGACAG |
| *Ccnb1*_Forward | AAGGTGCCTGTGTGTGAACC |
| *Ccnb1*_Reverse | GTCAGCCCCATCATCTGCG |
| *Dgat2*_Forward | GCGCTACTTCCGAGACTACTT |
| *Dgat2*_Reverse  AAV-shCIDEC  AAV-shCREBH | GGGCCTTATGCCAGGAAACT  TCGTGGAGACAGAAGAATACTCGAGTATTCTTCTGTCTCCACGA  CGGAACAAACAGTCAGCTCAACTCGAGTTGAGCTGACTGTTTGTTCCG |
|  |  |

**References:**

1. M.W. Lee, D. Chanda, J. Yang, et al., "Regulation of hepatic gluconeogenesis by an ER-bound transcription factor, CREBH," *Cell Metab*, 11, (2010): 331.

2. K.H. Song, A.Y. Park, J.E. Kim, et al., "Identification and characterization of cyclic AMP response element-binding protein H response element in the human apolipoprotein A5 gene promoter," *Biomed Res Int*, 2013, (2013): 892491.

3. C. Zhang, G. Wang, Z. Zheng, et al., "Endoplasmic reticulum-tethered transcription factor cAMP responsive element-binding protein, hepatocyte specific, regulates hepatic lipogenesis, fatty acid oxidation, and lipolysis upon metabolic stress in mice," *Hepatology*, 55, (2012): 1070.

4. J.H. Lee, P. Giannikopoulos, S.A. Duncan, et al., "The transcription factor cyclic AMP-responsive element-binding protein H regulates triglyceride metabolism," *Nat Med*, 17, (2011): 812.

5. X. Xu, J.G. Park, J.S. So, et al., "Transcriptional regulation of apolipoprotein A-IV by the transcription factor CREBH," *J Lipid Res*, 55, (2014): 850.

6. H. Kim, R. Mendez, Z. Zheng, et al., "Liver-enriched transcription factor CREBH interacts with peroxisome proliferator-activated receptor alpha to regulate metabolic hormone FGF21," *Endocrinology*, 155, (2014): 769.

7. X. Xu, J.G. Park, J.S. So, et al., "Transcriptional activation of Fsp27 by the liver-enriched transcription factor CREBH promotes lipid droplet growth and hepatic steatosis," *Hepatology*, 61, (2015): 857.
